# Utilisation of Inorganic Phosphates in Standard Diets for Whiteleg Shrimp *Litopenaeus vannamei* (Boone, 1931)

**DOI:** 10.3390/ani15192769

**Published:** 2025-09-23

**Authors:** Yosu Candela-Maldonado, Raquel Serrano, Ana Tomás-Vidal, David S. Peñaranda, Ignacio Jauralde, Laura Carpintero, Juan S. Mesa, José L. Limón, Javier Dupuy, Andrés Donadeu, Guillermo Grindlay, Judit Macías-Vidal, Silvia Martínez-Llorens

**Affiliations:** 1 Aquaculture and Biodiversity Research Group, Institute of Science and Animal Technology (ICTA), Universitat Politècnica de València, 46022 Valencia, Spain; yocanmal@upv.edu.es (Y.C.-M.); atomasv@dca.upv.es (A.T.-V.); dasncpea@upv.es (D.S.P.); igjaugar@doctor.upv.es (I.J.); 2 Department of Analytical Chemistry, Nutrition and Food Sciences, University of Alicante, P.O. Box 99, 03080 Alicante, Spain; raquel.serrano@ua.es (R.S.); guillermo.grindlay@ua.es (G.G.); 3 R&D Department, Global Feed, S.L., Tervalis Group, Av. Francisco Montenegro s/n, 21001 Huelva, Spain; laura.carpintero@tervalis.com (L.C.); juan.sebastian@tervalis.com (J.S.M.); joseluis.limon@tervalis.com (J.L.L.); javier.dupuy@tervalis.com (J.D.); andres.donadeu@tervalis.com (A.D.); judit.macias@tervalis.com (J.M.-V.)

**Keywords:** inorganic phosphorus source, *Litopenaeus vannamei*, digestibility, P and N excretion

## Abstract

Aquaculture effluents rich in phosphorus and nitrogen (P and N) can lead to eutrophication of aquatic ecosystems. These nutrients may contribute to harmful algal blooms, oxygen depletion, and deterioration of water quality, which poses a threat to aquatic biodiversity. In shrimp diets, environmental impacts from P and N nutrient leaching can be reduced by improving dietary P digestibility through the use of alternative ingredients. While fishmeal, with its high phosphorus content, has traditionally been a primary source, its declining use due to cost and limited availability necessitates the inclusion of inorganic P sources to meet shrimp nutritional requirements. Optimising these sources ensures adequate phosphorus availability while minimising nutrient waste. This study evaluated the effects of inorganic phosphate supplementation (monoammonium phosphate, MAP; monosodium phosphate, MSP; and sodium calcium phosphate, SCP-2%) in standard diets (35% CP) on nutrient digestibility, residue generation, and performance of *Litopenaeus vannamei*. Results showed that phosphorus digestibility exceeded 96% across all phosphate sources, with MSP achieving the highest values. Calcium digestibility was notably higher in diets containing monocalcium phosphate, such as SCP-2%, which demonstrated superior digestibility values. No significant differences were observed in nitrogen or phosphorus excretion; however, residue analysis revealed that SCP-2% diets generated the lowest nitrogenous waste relative to ingested nitrogen, whereas MAP diets produced the highest nitrogen residues, followed by the Control diet. For phosphorus residues, the Control diet showed the greatest proportion relative to ingested phosphorus, followed by MSP. Phosphate inclusion did not affect shrimp growth, survival, or body composition. However, phosphorus and calcium retention efficiencies were inversely proportional to their dietary content, underscoring the importance of optimising phosphate sources to enhance nutrient utilisation and minimise environmental impact.

## Simple Summary

This study examined the impact of inorganic phosphate additives on nutrient digestibility, waste generation, and performance of *Penaeus vannamei*. Regarding to P digestibility, monosodium phosphate (MSP) showed the highest values. SCP-2% (sodium calcium phosphate with 2% sodium incorporation) diet showed the highest values of Ca digestibility. Although nitrogen and phosphorus excretion did not differ significantly, residue analysis showed that SCP-2% diet produced the lowest nitrogen waste, while monoammonium phosphate (MAP) and the Control diet generated the highest levels. Phosphorus residues were greatest in the Control diet, followed by MSP. Overall, phosphate inclusion did not influence shrimp growth, survival, or body composition, but phosphorus and calcium retention declined as their dietary levels increased, highlighting the need to optimise phosphate sources for improved nutrient efficiency and reduced environmental impact.

## 1. Introduction

*Litopenaeus vannamei* is the predominant shrimp species cultivated in the world, with a total production of almost 7 million tons in 2024 which is equivalent to U$D 32 million [1], accounting for a substantial portion of aquaculture production due to its good growth and high profitability. *Litopenaeus vannamei* was selected as the experimental species due to its global importance in aquaculture and its comparatively lower dietary protein requirements relative to other shrimp species. This characteristic makes it a suitable model for nutritional studies focused on sustainable aquafeeds [2].

Although shrimp production has greater potential, supplying of high-quality feed ingredients for shrimp aquaculture is one of the major challenges. Diets with proper nutrient balance are important in enhancing shrimp health and higher production. Phosphorus (P) is an essential nutrient in shrimp diets, playing a vital role in supporting the overall growth, development, and health of aquatic species such as *Litopenaeus vannamei* [3]. This mineral is particularly critical for the formation and maintenance of the shrimp’s exoskeleton, which serves as a protective structure and aids in moulting processes necessary for growth. Additionally, P is a key component in cellular energy metabolism, facilitating the conversion of energy through adenosine triphosphate (ATP) and adenosine diphosphate (ADP) pathways, which are fundamental to various biological processes. P also functions in acid-base buffering within the blood, helping to maintain physiological balance, and is an integral part of tissue cell membranes, contributing to the structural integrity and function of cells [4].

Traditionally, shrimp diets have relied on mineral-rich ingredients, such as fishmeal (FM), to supply adequate levels of bioavailable P. However, the increasing shift toward sustainable aquaculture has led to a reduction in fishmeal content in commercial diets. These modern diets often include alternative plant-based or other protein sources, which are typically lower in available phosphorus. As a result, shrimp fed diets may not meet their P requirements, potentially leading to reduced growth performance and compromised health [5,6,7,8]. This challenge has prompted the need for supplementation with inorganic P sources or the exploration of other strategies to ensure adequate P levels in aquaculture diets. Commercial shrimp diets typically contain between 1 and 1.5% of total phosphorus (P) for optimal juvenile growth and development. This requirement can vary based on shrimp size and other factors ranging between 0.8 and 2%. Research suggests that the phosphorus requirement in diets with low content of fish meal for *L. vannamei* ranges from 1.12% to 1.27% to enhance the immune response and antioxidant capacity and the disease resistance, respectively [9]. The nutritional composition of such diets is particularly crucial, as shrimp increasingly derive a larger proportion of their nutritional requirements from these artificial diets [3].

There is increasing awareness about the environmental impact generated by effluents from shrimp farming. As can be seen in a study carried out by Dinesh Kumar providing a detailed analysis of nitrogen (N) and P content in wastewater from *L. vannamei* farms along the southeast coast of India [10]. Phosphorus, a crucial nutrient released into the environment through shrimp culture effluent, has been identified as a major contributor to coastal eutrophication due to its scarcity in natural aquatic ecosystems in shrimp farms [10]. These nutrient concentrations in the effluents were reported to range between 0.5 and 2.8 mg/L (P levels) [11] and 8.7 and 21.5 mg/L (N levels) [9,11]. Elevated phosphate levels contribute to the risk of eutrophication, promoting harmful algal blooms and degrading water quality in receiving ecosystems. High total N levels, particularly in forms such as ammonia and nitrate, exacerbate oxygen depletion in water bodies, threatening aquatic biodiversity.

Thus, selecting an appropriate P source is vital for enhancing P bioavailability to cultured shrimp, reducing unnecessary phosphorus supplementation, and minimising nutrient waste in both the culture system and the effluent waters [11,12], making it a key factor in the development of artificial diets. Future research should focus on identifying high-quality P sources that are well-suited for different species of shrimp at various stages of development. These studies will be critical for producing diets that are both economically viable and ecologically responsible, ultimately supporting the long-term sustainability of shrimp aquaculture.

For an inorganic phosphorus source to be considered ideal, it must meet specific criteria; these include high solubility, which directly correlates with high digestibility; a low calcium-to-phosphorus (Ca/P) ratio, ideally less than 1 to enhance phosphorus absorption [7]; and a low buffer capacity, which facilitates digestion by reducing the energy expenditure required to achieve optimal pH conditions during the digestive process [13].

Among the inorganic phosphates available on the market, tricalcium phosphate (TCP) and dicalcium phosphate (DCP) exhibit the lowest solubility [14] and, consequently, the lowest digestibility in species like fish and shrimp. On the other hand, monosodium phosphate (MSP) [15] offers excellent solubility and high bioavailability, but its use is often limited by its high cost. Monoammonium phosphate (MAP), another commonly used phosphorus source, also shows good availability [14]; however, its ammonia content poses environmental concerns, as it can be excreted into surrounding waters, contributing to nutrient pollution [16]. Monocalcium phosphate (MCP), despite its relatively high calcium-to-phosphorus ratio and significant buffer capacity [17], is still widely used due to its bioaccessibility. However, a high buffer capacity implies that more energy can be required by the shrimp to maintain optimal digestive pH, reducing overall energy efficiency.

In response to these limitations, new formulations of advanced inorganic phosphates are under development. One such innovation is sodium calcium phosphate, SCP-2%, a monobasic phosphate that fulfils all the criteria for an ideal inorganic phosphorus source: high solubility, a low Ca/P ratio, and low buffer capacity, making it an optimal choice for enhancing P absorption [16,17]. Despite the positive aspects of SCP-2%, this P source has not been tested before in shrimp diets.

Thus, it is critical to evaluate the digestibility and retention of different inorganic P sources to meet the specific P requirements of shrimp without excessive supply, which could otherwise lead to increased P excretion and subsequent environmental degradation [10].

The determination of P sources and their bioavailability in diets for *Litopenaeus vannamei* is of paramount importance, given the environmental impact associated with excessive phosphorus discharge. However, evaluating the bioavailability of N or P requires precise methodologies to accurately quantify their utilisation and assimilation within the organism.

Thus, in the present study, three inorganic phosphates were compared to select the optimal to evaluate different P sources (monosodium phosphate, MSP; monoammonium phosphate, MAP; and monosodium/monocalcium phosphate, SCP-2%), considering both nutritional and environmental perspectives by performing digestibility and growth trials in shrimp fed practical diets.

## 2. Materials and Methods

### 2.1. Diets Formulation

Four practical diets were formulated to assess the P use containing 10% of fishmeal (Table 1). Testing three P sources: monoammonium phosphate (MAP, NH_4_H_2_PO_4_; Yara S.L., Helsingborg, Finland), monosodium/monocalcium phosphate (SCP-2%, NaH_2_PO_4_/Ca(H_2_PO_4_)_2_·H_2_O in proportion 12/88; AQphos+, Global Feed, S.L.U., Huelva, Spain), and monosodium phosphate (MSP, NaH_2_PO_4_; Global Feed, S.L.U., Huelva, Spain), plus a Control diet without P supplement. The P level in the basal diet (Control) to determine digestibility was 0.66% following the recommendations made as described in the literature [14].

The remaining experimental diets were supplemented with different inorganic phosphate sources to achieve a phosphorus level of 1.16% in the final feed. The MAP diet included 18.9 g/kg of monoammonium phosphate, while the SCP-2% diet, containing 2% sodium, used 21.4 g/kg of a monosodium/monocalcium phosphate blend. The MSP diet, with a higher sodium content (16.7%), included 20.8 g/kg of monosodium phosphate to reach the same phosphorus target. Wheat inclusion (35.8–37.15%) was adjusted depending on the phosphate source used. All diets were also supplemented with lysine and methionine to meet minimum levels of 2.0% and 0.8%, respectively, of these essential amino acids [18].

The experimental diets used in this work were manufactured by the cooking extrusion process at the Feed Manufacture of the Institute of Animal Science and Technology at the Universitat Politècnica de València (Valencia, Spain). A semi-industrial extruder from Clextral model BC45 (Saint Etienne, France) was used for this purpose.

The final composition of the diets is shown in Table 2, where the phosphorus (P) and nitrogen (N) levels closely matched the values predicted during formulation (Table 1). However, the MAP diet showed a higher analysed N content than expected, with values between 0.38% and 0.51% greater than those of the other diets. When expressed as crude protein (CP), this difference corresponds to an increase of 2.43% to 3.23% CP. This excess nitrogen is attributed to non-protein nitrogen, which should have been accounted for during the formulation process.

### 2.2. Experimental Design and Facilities

Growth trial, digestibility trial, and estimation of N and P trial were all performed in the facilities of the wet Aquaculture Laboratory from the Universitat Politècnica de València. Shrimp from these experiments were kept in an indoor RAS system conformed by tanks with a water volume of 90 L each. The system has a water flow of 40% per hour; water was purified using a mechanical filter, a foam fractionator, and a biofilter for the ammonia nitrogen oxidation.

#### 2.2.1. Digestibility Trial

The apparent digestibility of dry matter, protein (estimated as 6.25 × %N), Ca, and P of the four experimental diets was determined by incorporating Yttrium (Y_2_O_3_) (Sigma-Aldrich, Merck KGaA, Darmstadt, Germany) as an inert marker into the diet (Yttrium, Y), which was added to the mixtures shown in Table 1 at 5 g/kg.

The digestibility trial was conducted in tanks with a capacity of 90 L, for the time required to obtain sufficient faeces to perform the analyses of Y, N, P, and other nutrients (15–21 days), in parallel with the growth trial. A 4 × 4 × 4 Latin square design, consisting of four diets, four tanks, and four replicates, was applied. Approximately 30 shrimp, with an average body weight of 15.0 ± 0.1 g, were placed in a mesh cage within each tank. The digestibility trial commenced after five days of acclimatisation to the recirculating system, with the aim of collecting the necessary amount of faeces for the determination of yttrium, nitrogen, calcium, and phosphorus.

**Figure 1:**
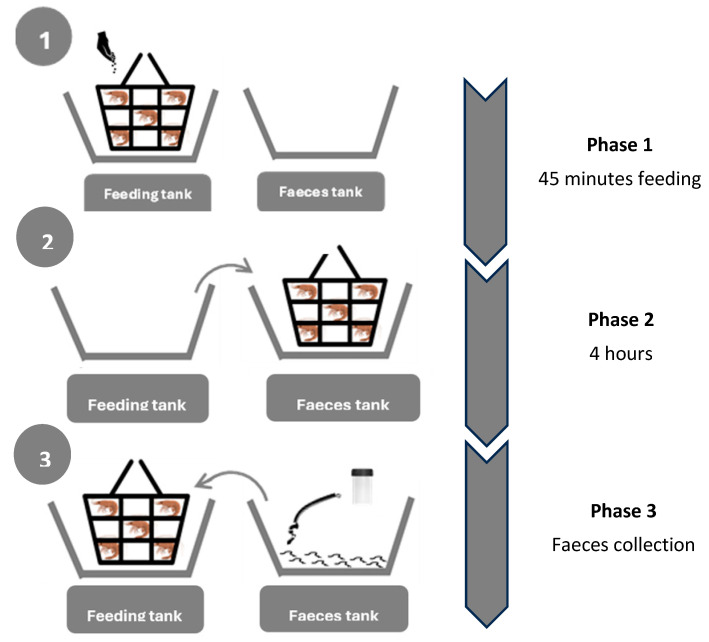
Illustration of the faeces collection system used in the digestibility trial. As can be seen, 3 phases are described. In phase 1, shrimp are fed in the feeding tank, where they will remain for 45 min. In phase 2, shrimp are moved into the faeces tank, where they will remain for 4 h. In phase 3, shrimp are replaced into feeding tank, leaving faeces intact in faeces tank ready to be collected by syphon.

The collection of faeces was carried out using a new syphon collection system (Figure 1). It consists of a RAS system of clear water tanks divided into compartments. Each compartment consisted of two tanks: the feeding tank within the recirculating system, where the shrimp were fed, and another tank, the faeces tank outside the recirculating system, containing clean water and used for faeces collection. All tanks had a capacity of 90 litres each. During the experiment, 30 shrimp were placed inside a mesh cage. Firstly, shrimp cage was placed inside the feeding tank at the time of feeding the shrimp. After 45 min in the feed tank, the cage was moved to the faeces tank where the shrimp remained for 4 h, collecting faeces every hour.

After this period of time, the shrimp were returned into the feeding tank so that the faeces could be collected. This way, the rest of the faeces samples can be easily collected by syphon. Due to the use of two different tanks (faeces tank and feeding tank), this system allowed to separate faeces sample from uneaten pellets or other shrimp residues generated during feeding. Therefore, during this experiment, there was no risk of contamination in the collected sample.

The health status of the shrimp was monitored daily, and they were fed at a restricted feeding rate (2.7 g/100 g of shrimp per day, for a water temperature between 27.3 and 28.1 °C), twice per day, at 8 a.m. in the morning and at 5 p.m. in the afternoon. 45 min after feeding, the animals were transferred using a closed basket to a “clean” tank where there were no remains of feed to avoid contamination of the faeces with the diet, and the faeces were collected by syphon every hour for four hours when the shrimp were returned to the recirculation system.

Similarly, the water physical–chemical parameters, such as temperature, oxygen, and nitrogenous compounds (ammonium, nitrites, and nitrates), were controlled daily that resulted in the optimal development of the species: T 27.7 °C; pH 7.92; O_2_ 6.36 mg/L; salinity 20 mg/L; and NO_3_^−^ 26.3 mg/L. Ammonia and nitrite levels were lower than the detection limit of the method throughout the experiment (N-NO_2_-/N-NH_4_^+^ <0.001 mg/L). Once collected, the faeces were frozen and lyophilized prior to analysis.

#### 2.2.2. Growth Trial

Shrimp growth trial was performed in the facilities of the wet Aquaculture Laboratory from the Universitat Politècnica de València. Shrimp used for this study came as larvae from White Panther hatchery in Austria. Shrimp were placed in 16 (4 tanks per treatment) plastic rectangular tanks with a capacity of 90 L each, with independent and constant aeration and temperature regulation. Twenty shrimp were placed in each tank.

Experiment began with shrimp having an initial average weight of 0.5 ± 0.01 g per animal and lasted 96 days of feeding until commercial weight. Daily intake calculations were based on an optimal daily feeding rate (DFR) proposal, considering shrimp weight and average water temperature [19]. Feeding took place twice a day at 8 a.m. in the morning and at 3 p.m. in the afternoon, as recommended by [3]. Every three weeks, all the shrimp from each tank were sampled to assess growth and recalculate feed intake. Given that the temperature throughout the trial was 27.7 °C, feed intakes were recalculated weekly using the average water temperature and the possible weekly biomass increase based on previous sampling growth data.

### 2.3. Water Quality Analysis

Water quality parameters, including temperature, oxygen levels, and N compounds (ammonia, nitrites, and nitrates), were monitored daily. These parameters remained within the optimal range for the species’ development: pH 7.92, O_2_ 6.36 mg/L, salinity 20 mg/L, and NO_3_^−^ 26.3 mg/L. Ammonia and nitrites were lower than the detection limit of the method throughout the growth experiment (N-NH_4_^+^ < 0.001 and N-NO_2_^−^ < 0.001 mg/L, respectively). The source of seawater used in the experiments was transported from outside Valencia’s port.

### 2.4. Estimation of N and P

The determination of N and P excretion was carried out in the same system where the digestibility and the growth trial were performed. It consisted of a RAS system of clear water tanks divided into compartments, a total of 4 compartments and 8 tanks (2 tanks per compartment and 1 compartment per treatment). Each compartment consisted of 2 tanks (a feeding tank and an excretion tank) of 90 litre capacity each. Ten shrimp were initially placed in the feeding tanks. Half an hour after feeding, the shrimp will be placed in the excretion tanks where they will remain for the rest of the experiment and where water samples will be collected. Feeding was calculated considering shrimp weight and average water temperature [19].

The sampling and analysing soluble N and P water concentration was carried out in a 24 h cycle by quadruplicated tanks per diet. It consisted of feeding the shrimp daily at 8.00 a.m. at a restricted feeding rate, which was established based on the average weight of the shrimp, the temperature of the water, and the biomass of the tank (see digestibility section). Water samples were taken one hour before and after feeding and every two hours thereafter. The ammonia excretion was established for the different experimental groups. Analyses were carried out following the method detailed in McGoogan and Gatlin [20]. The pump was turned off during the entire ammonia sampling period, altering the recirculating nature of the system in order to estimate the ammonia increase due to excretion. In addition to collecting water samples from the tank, the established parameters (pH, temperature, and dissolved oxygen) were also measured.

### 2.5. Analysis and Calculations

Chemical analyses were conducted at the Laboratory of Animal and Technology Science Institute at Universitat Politècnica de València. Analyses included raw material, diet composition, faeces, and shrimp. After determining dry matter, the samples were lyophilized for subsequent analysis, following AOAC procedures [21]: dry matter (105 °C to constant weight), ash (by incineration at 550 °C to constant weight), and protein (by the Dumas method, converting all N forms to N gas through combustion and measuring by thermal conductivity using a LECO CN 628 protein analyser). Determination of Ca and P: all samples were digested in triplicate at the University of Alicante with a high-pressure microwave assisted digestion system (UltraCLave Milestone s. r. l., Sorisole, Italy). To do that, 4 mL of 67% w w^−1^ HNO_3_ was added to 0.10 g of the lyophilized samples in Teflon vessel. Then, samples were heated at 240 °C and maximum power during 30 min. Then, samples were brought to a final weight of 15 g with ultrapure water and finally filtered using a syringe filter of 0.45 µm pore size. Samples were stored in polyethylene vials at 4 °C until analysis. Ca and P were quantified in the solution using Mas Inductively Coupled Plasma Mass Spectrometry (ICP-MS).

The apparent digestibility coefficients for dry matter, protein, Ca, and P will be calculated as:

ADC (%) = 100 × [1 − (Nh/Np × DY/HY)]

where Nh is the percentage of protein, calcium, or the amount of phosphorus in the faeces and Np is the percentage of protein, energy, or the amount of phosphorus in the diet. DY is the concentration of Yttrium in the diet, and HY is the concentration of Yttrium in the faeces [22].

The P and Ca digestibility of each inorganic phosphate was determined by the following equation [14]:

ADC phosphorus (inorganic phosphate) = [(A + B) × C − (A × D)]/B

where A: Contribution of P in the Control diet to the nutrient content of the experimental diet. In the case of Control diet, 0.66% comes from 0.39% of the fishmeal and 0.27% of the rest of the ingredients. B: Contribution of P in the inorganic phosphate (SCP-2%, MAP or MSP) to the P content of the experimental diet, which is 0.5%, which represents 43.3% of P contribution in inorganic form. C: ADC of P in the experimental diet (SCP-2%, MAP, or MSP). D: ADC of P in the Control diet.

The method used for the determination of ammonium in water was Grasshoff’s method [23]. This method measures all ammonia nitrogen (N-NH_3_ and N-NH_4_^+^).

To determine the soluble P (orthophosphates), the sample must be filtered beforehand. Both soluble and total P were determined with the help of a multiparametric photometer following Baumgarten manual of water analysis [24].

Phosphorous and nitrogen total wastes were calculated considering N and P, ingested and digested, as well as N and P retained, following the nutrient mass balance for N and P proposed by Morales et al. [25].

Nutrient Retention Efficiency (RE) was calculated as:

RE = 100 × ((BW_final_ × N_final_) − (BW_inicial_ × N_inicial_)) × (FCR × (BW_final_ − BW_inicial_) × N_diet_)**^−^**^1^

where N_diet_ is the content of nutrient in the diet, and N_initial_ and N_final_ represent the initial and final concentration of nutrient in whole minced shrimp.

Solid nitrogen (N) or phosphorus (P) discharges were calculated as:

Solid Nutrient Discharge = N or P intake (100 − ADC of N or P) × 100^−^^1^

Dissolved N or P discharge was calculated as:

Dissolved Nutrient Discharge = (N or P intake × ADC 100^−^^1^) − (N or P retained in whole body shrimp)

### 2.6. Statistical Analysis

Growth data, nutrient data, biometric parameters, body composition, excretion data, protein and fat retention, and digestibility data were treated using analysis of variance (ANOVA). The Student-Newman-Keuls test was used to evaluate specific differences between diets. Data were considered statistically significant when *p* < 0.05, and data are shown as the mean with standard error of the mean (±SEM). Statistical analyses of the data were performed with Statgraphics, Statistical Graphics System, version Centurion 18-X64.

## 3. Results

### 3.1. Digestibility Coefficients

The results for the digestibility trial are shown in Table 3. The digestibility coefficients of dry matter, as well as protein, did not present statistically significant differences in any of the treatments.

P digestibility of shrimp fed diet without phosphates (Control diet) presented the lowest (75.4%) than the rest of the diets containing inorganic phosphates, where its P digestibility ranged between 84.3 and 86.0%, without significant differences among them.

However, when P digestibility of the inorganic phosphates was calculated (Table 3), it was observed that MSP phosphate exhibited the highest P digestibility (100%). MAP and SCP-2% did not present significant differences between them (96.1%).

### 3.2. Growth and Nutritional Parameters

Figure 1 illustrates the average weight evolution of shrimp fed with the four experimental diets over the 96-day period. In general, shrimp growth was high throughout the trial, regardless of treatment type. SCP-2% showed a clear tendency as the lower final weight treatment over time; however, no significant differences were found among treatments.

Growth and nutritional parameters for the shrimp at the end of the growth trial are presented in Table 4. Survival rates in all treatments were high and were not significantly affected by diets (*p* < 0.05). Similarly, growth parameters, including final weight and feed conversion ratio (FCR), were not influenced by the diet, achieving an average shrimp final weight of 13 g.

No significant differences were observed among treatments concerning body composition and N retention efficiency (NPV) (Table 5). Only the efficiency of P and Ca retention showed significant variation among diets, being lower in shrimp fed with diets containing higher levels of both minerals.

### 3.3. Phosphorus and Nitrogen Excretion Results

**Figure 2:**
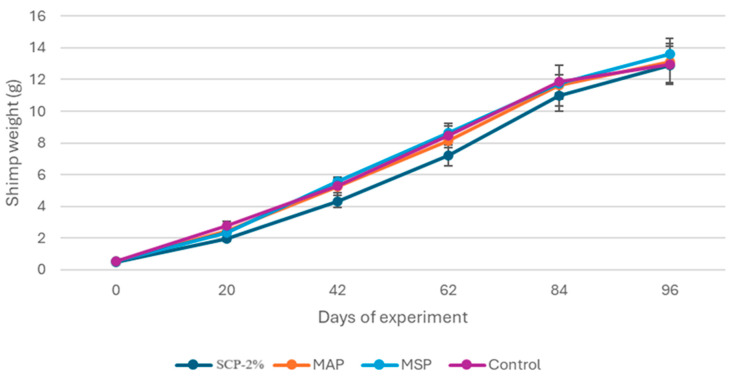
The data in the graphic show the shrimp weight increase (g) mean of four tanks (*n* = 4) ± standard error of the mean (ES). No significant differences were observed in any sampling (*p* < 0.05). Newman-Keuls test.

Figure 2 shows the postprandial excretion of both total ammonia nitrogen (N-NAT) and soluble phosphorus (P-PO_3_^4−^) of shrimp fed the different diets. The peak of excretion occurred between 4 and 8 h post-feeding, although this was much more dispersed in the case of P than in N excretion. Regarding N excretion, this was the highest in shrimp fed with MAP diet feeds at 4 h postprandial.

**Figure 3:**
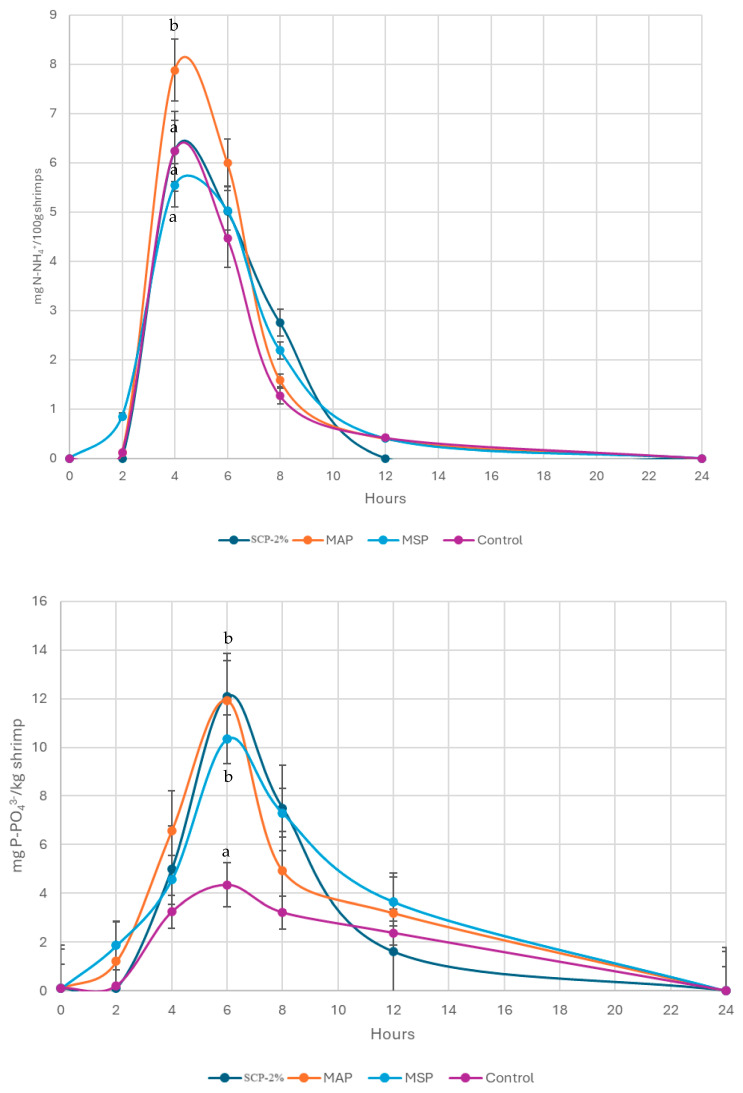
Ammoniacal excretion is expressed as total ammoniacal nitrogen (N-TAN) and phosphorus (P-PO_4_^3−^), expressed in mg of N/100 g of shrimp and hour and P in mg of N/kg of shrimp and hour, respectively, in each of the treatments. The data in the graphs show the mean of four tanks (*n* = 4). Hour 0 was considered the time when shrimp ceased feeding. Different letters at each hour indicate significant differences among diets (*p* < 0.05). Newman-Keuls test.

Phosphorous excretion peaked at 6 h postprandial (Figure 3). As it was expected, the lowest peak was observed in shrimp fed with Control diet, the diet with the lowest P intake. The rest of the treatments that included inorganic phosphates did not present relevant differences in terms of P excretion.

In Table 6, the daily N and P excretion of shrimp is shown, obtained as an average of 4 days along 24 h of measurements in each tank.

The excretion of N or P did not present significant relevant differences, but when P and N excretion and retention were evaluated jointly with P and N digestibility, the SCP-2% diet generated the lowest N waste in relation to the N intake. The MAP diet presented the highest amount of N residues due to N excretion, which was greater than in the rest of the diets, followed by the Control diet.

**Figure 4:**
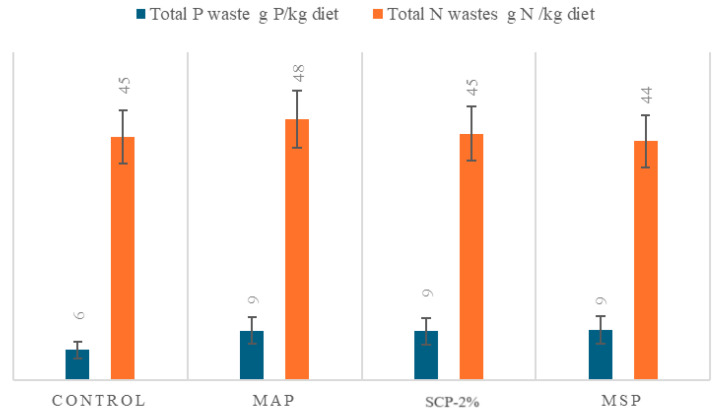
Total P and N waste generation expressed P and N wastes per kilogram of diet ingested considering the N and P waste produced from faeces, soluble wastes of excretion, and P and N retention.

The total N and the P waste generation expressed in grams of P or N per kg of ingested diet is shown in Figure 4, where the sum of waste produced from faeces, soluble waste from excretion, and N and P retention has been considered and expressed N and P wastes per kilogram of diet ingested. MAP diet exhibited the highest waste generation in the case of N, although no significant differences were observed among treatments. As it was predictable, Control diet showed the lowest P waste generation.

## 4. Discussion

Findings from the present study note the suitability of use of monobasic inorganic phosphates in diets for *Litopenaeus vannamei*, reporting an improvement of P utilisation in standard shrimp diets. These findings are consistent with previous studies, highlighting the effectiveness of different inorganic P sources, such as monosodium phosphate (MSP), monoammonium phosphate (MAP), and monocalcium phosphate (MCP) yielded high phosphorus digestibility in *L. vannamei* diets, particularly when these inorganic sources were compared with magnesium phosphate [26]. However, in the present study, we also evaluated other inorganic phosphates, including SCP, following the chemical properties of this phosphate source, like its low buffer capacity. The buffering capacity of inorganic phosphates may influence the pH of the digestive content, thereby modulating the activity of digestive enzymes. Such modulation can enhance the efficiency of nutrient hydrolysis, ultimately leading to improved protein utilisation and mineral availability [16]. Consequently, due to the inclusion of inorganic phosphates with an appropriate buffering capacity in shrimp diets could contribute not only to optimising digestive processes but also to increasing the overall protein and mineral content assimilated by the organism, the SPC-2% was included in the present study.

Additional point on high phosphorus digestibility observed in present study may be explained in Ca/P, as has already been noted in previous studies [14], as low Ca dietary improves the P absorption through intestine. In the present experiment, the formulation of diets with a constant Ca/P ratio was not feasible, since these compounds inherently differ in this parameter, among other physicochemical aspects. In fact, the Ca/P ratio is one of the key criteria used to classify and distinguish the various types of inorganic phosphates. This classification is not arbitrary but is officially recognised and standardised in regulatory frameworks, as exemplified in the European Union Commission Regulation (EU) 2017/1017 of 15 June 2017, amending Regulation (EU) No 68/2013 on the Catalogue of Feed Materials (Off. J. Eur. Union 2017, 159, 48–119) [27]. As illustrated in this regulation [16,17], the variability of the Ca/P ratio is intrinsic to the identity of each phosphate source; therefore, it cannot be homogenised across treatments without altering the nature of the materials under study.

Optimal levels of P in the shrimp diet can be strongly affected by the Ca/P ratio of the shrimp, as well as the individual level of minerals [28,29]. An excess of Ca can negatively affect P utilisation, probably due to the formation of insoluble complexes in the intestinal lumen that reduce P absorption. High Ca levels relative to P reduce phosphorus absorption, leading to deficiencies that can impair growth, reduce feed efficiency, and weaken the exoskeleton [28]. This weakening increases susceptibility to injuries and infections [26]. Similarly, low calcium levels in a high-P diet may not meet the requirements for cellular functions, potentially resulting in metabolic disturbances [30]. Therefore, ensuring that the ratio of these two elements is balanced is important for shrimp nutrition. In the case of crustaceans, the Ca/P ratio in diet is considered important, as it has been implicated in improving growth and preventing structural deformities such as the soft carrycot. It is recommended that Ca/P be maintained at 1 or even less in diets for *L. vannamei*, depending on water salinity, to maintain normal growth of the shrimp [30]. In any case, diets with more than 2% Ca, because they reduce growth, are not recommended. The literature suggests that diets which contained a Ca/P ratio of 1 produced a 3-fold increase in weight gain and a good recovery in soft-shelling compared to diets without supplemental Ca or P [3]. However, in the present experiment, the Ca/P ratio did not exceed 1% in any of the treatments. The observed values ranged from 0.49 in the MSP-based diet to 0.72 in the SCP-2% diet, both of which were lower than the ratio recorded in the Control diet (0.81). These differences in Ca/P ratios largely reflect the intrinsic Ca/P proportions of the specific inorganic phosphate sources incorporated into each dietary treatment, as shown in Table 2. Therefore, no negative effects on P absorption have been observed for this reason, nor due to the differences observed in the different diets. Similar Ca/P ratios were used in recent studies of inorganic phosphates supplementation, 0.32–0.90 [14] and 0.38–0.47 [26]. Regarding the Control diet, a greater P absorption in diets containing inorganic phosphates was shown when digestibility was calculated separately. Thus, it can also be seen that MSP phosphate presented the highest digestibility of P, with significant differences between the P of MAP and SCP-2% phosphates.

Therefore, the selection of P sources should consider not only the Ca/P balance but also the potential environmental impacts. Controlled supplementation that provides an adequate Ca/P ratio without exceeding P needs supports optimal shrimp performance and reduces the risk of eutrophication in pond systems [3].

In general terms, the digestibility of Ca was much lower than that of N or P, as it was expected. However, the digestibility of Ca in the diet containing inorganic calcium, such as SCP-2%, was significantly higher than in the rest of the diets. The high Ca digestibility of this phosphate was observed by calculating the Ca digestibility in each of them which, in the case of SCP-2% phosphate, was 93.1%. Control diet, followed by MAP and MSP, reported lower digestibility of this nutrient than SCP-2%. Similar results were seen [26] with diets containing monocalcium phosphates showing the highest Ca digestibility, attributing this fact to the synergistic interaction between Ca and P, which likely enhanced mineral bioavailability. These results underscore the importance of selecting phosphate sources that optimise both Ca and P bioavailability in shrimp diets, thereby supporting the improvement of mineral utilisation and overall growth performance [3,30].

Ca, as well as P, Mg, K, and 11 other minerals are essential nutrients for shrimp [3,31], and like all species of crustaceans, shrimp have an additional mineral requirement due to the growth-related moulting process [32]. Studies in *Litopenaeus vannamei* with these macrominerals suggest the possibility that the requirement of Ca and Mg is met by absorption of the surrounding water under typical conditions of high marine salinity [33], although dietary supplementation may be necessary in shrimp produced in oligohaline medium. Even so, although few studies show a low or even negative digestibility of this nutrient [14], in MSP and SCP-2% phosphates there was a high absorption of this nutrient, which should be considered in supplementing these additives, especially in waters with low alkalinity where Ca is in low concentrations.

Although phosphorus requirements in crustaceans are not well-defined, the estimations for *L. vannamei* range from 0.34% to 2.2%, and for penaeids in general, phosphorus requirements are estimated from dietary supplementation range between 1% and 2%. It is clear that phosphorus needs vary significantly with Ca supplementation [28]. In this study, P was supplemented through various inorganic forms in the diet at 0.5%, with an additional 0.66% from other ingredients (1.16% total P in the diet). Thus, we consider that the total P requirement of the shrimp was covered with the experimental diets, except for the Control diet which did not include any inorganic phosphate. Nonetheless, no differences in growth were observed between Control and other diets. Previous studies have found that this P level (1.16%) meets the species requirement [14]. Certain authors [7] observed that supplementation with inorganic P (e.g., sodium phosphate) was ineffective in inducing a weight gain response with shrimp fed diets containing from 1% to 2.5% of dietary P (without Ca supplementation, containing 0.5% Ca), showing similar positive growth. Although they noted lower growth in the control diet, which was not supplemented with phosphate (0.5% P), they also showed that increasing dietary Ca levels raised P needs for optimal growth and that excessive Ca depressed shrimp growth, even with increasing P levels. This is consistent with other studies [28,29], which indicate that high Ca content in the diet can inhibit P and other nutrient absorption, suggesting that Ca content should be reduced in shrimp diets. In our study, however, despite some phosphates containing Ca, the total dietary Ca did not exceed 1%; therefore, there were not significant negative effects on shrimp growth.

Although the Control diet in the present study did not cover the nutrient P requirements, no significant differences were reported in shrimp growth. Similar results can be seen in other studies in which, with 1.1% phosphate supplementation in the diet, they obtained a significantly better growth compared to their control diet [12,26]. In contrast to our findings, a recent study [14] showed differences in shrimp growth between a control diet (0.5% P level, without P supplementation) and monobasic phosphate-supplemented diets (0.8% of P level). Likewise, other studies reported lower growth in a control diet with low P level (0.5%, similar to our Control diet, 0.66% P) when it was compared with the rest of experimental diets containing inorganic phosphate [7]. Their control diet was an experimental diet, difficult to compare to our practical shrimp diet, formulated with the aim to cover P levels below the shrimp’s needs. However, they concluded that shrimp fed P-limited diets (control and DCP) might recover minerals through exuvia ingestion, which could explain the similar growth of shrimp fed the control diet in the current study. Besides the effects on growth, deficiencies in certain minerals adversely affect the immune system and increase disease risk, which might lead to a low survival rate, as has been shown in diets with low P content [26]. However, the impact of mineral deficiencies on immunity and disease in shrimp is not well-studied, and it is complicated by their multiple mineral sources (diet and water), the effects of these sources, and interactions with other minerals and nutrients. Nevertheless, some studies have shown that low fishmeal levels (currently used in the shrimp industry) can reduce immune responses, which improve with phosphate supplementation. Certain literature suggested that a low fishmeal diet with 1.12% phosphorus improved immune responses, and that 1.27% P could enhance disease resistance and antioxidant capacity compared to a diet with 0.96% P (without affecting growth) [9]. This P level was higher than that in the Control diet used in this study. Thus, despite no observed differences in growth and survival, shrimp fed the low-P control diet might face challenges under large-scale production conditions, which were not evident in the controlled experimental setting.

Regarding retained P, higher levels were observed in the shrimp fed with MAP and SPC diets, suggesting a lower generation of P waste in the environment and a higher efficiency in nutrient utilisation than the other diets. No significant differences were observed between the various treatments concerning body composition and protein retention efficiency, a result consistent with similar previous studies [34]. Only the efficiency of P and Ca retention showed significant variation among diets, being lower in shrimp fed with diets containing higher levels of both elements. Between 11% and 13% of the P ingested was incorporated into the shrimp tissue, a result comparable obtained by Briggs and Funge-Smith [35]. However, in this particular study, nutrient retention in shrimp biomass was only 20–25% of N and 10–15% of P. Therefore, authors emphasised the need to progress on diet formulations and feeding strategies with the aim to enhance the nutrient utilisation and decrease waste, suggesting that better feed conversion ratios (FCRs) could mitigate nutrient losses [10].

Furthermore, although no statistically significant differences were found between the treatments, the animals fed with the MAP diet presented a higher excretion of N which is reflected in a greater generation of N residues (Figure 4). This effect has been observed in fish such as rainbow trout, which may be attributed to the fact that MAP contains NH_4_+, which is not retained in the animals’ bodies. The MAP nitrogen content might lead to an error when converting analysed N to crude protein [16]. Indeed, in the present experiment, the diet formulated with MAP contains a N content of 0.4% compared to the other diets, which do not include this supplement. This 0.4% increase results in elevated N excretion, as illustrated in Table 6 and Figure 4. In the context of the current experiment, this could extrapolate to an increase of 2.5–4 g/kg of feed entering the system compared to other diets. When applied to a production cycle, with feed conversion ratios in commercial farms ranging from 1 to 2, this could correspond to N discharges between 44 and 96 kg per ton of shrimp produced (values closely resemble to those obtained in other intensive commercial farm studies). Comparing MAP diet with other diets, this could result in a residue of up to 8 kg of N per ton of shrimp produced considering the maximum feed conversion ratio. This fact must be considered when establishing nutrient balance calculations.

**Figure 5:**
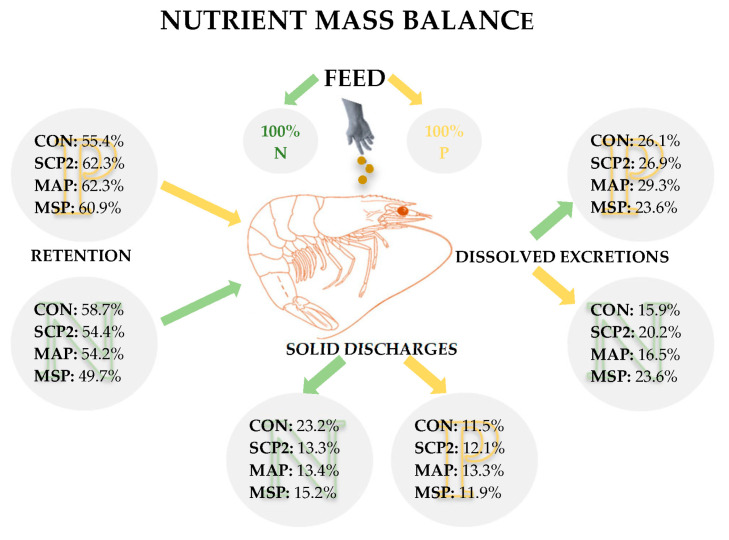
Total N and P budget regarding the N and P ingested calculated with results obtained from digestibility, excretion, and growth trial.

While the mass balance approach developed to estimate nutrient discharge into the aquatic environment does not allow for the distinction between nitrogen excreted through metabolic pathways (i.e., via branchial excretion) and unabsorbed, soluble ammonium ions derived from dietary MAP that traverse the gastrointestinal tract, the results of the present study indicate that MAP supplementation may be associated with a higher nitrogenous waste output compared to the other inorganic phosphorus sources assessed (Figure 5).

## 5. Conclusions

The inclusion of inorganic phosphates in shrimp diets significantly improved P digestibility, with values exceeding 96% across all phosphate sources. Calcium digestibility was particularly high in diets containing monocalcium phosphate, such as SCP-2%, which demonstrated good digestibility indices. While no significant differences were observed in N or P excretion among diets, residue analysis revealed notable trends: SCP-2% diet generated the lowest nitrogenous waste relative to ingested N, whereas the MAP diet produced the highest N residues, followed by the Control diet. In terms of P residues, the Control diet yielded the greatest proportion relative to ingested P, followed by the MSP diet. Importantly, the inclusion of phosphates did not affect shrimp growth, survival, or body composition, although P and Ca retention efficiencies were inversely proportional to their dietary content.

## Figures and Tables

**Table 1:** Ingredients and theoretical composition calculated from the analysed composition of the ingredients of the experimental diets.

INGREDIENTS (G/KG)	CONTROL	MAP	SCP-2%	MSP
FISHMEAL A	100	100	100	100
WHEAT MEAL B	399.0	369.0	365.0	366.0
SOYBEAN MEAL C	304.0	315.1	316.6	316.2
GLUTEN MEAL D	80	80	80	80
FISH OIL	20	20	20	20
SOYBEAN OIL	27	27	27	27
MALTODEXTRIN	50	50	50	50
MAP E		18.9		
SCP-2% F			21.4	
MSP G				20.8
VITAMIN PREMIX H	10	10	10	10
SOYBEAN LECITHIN ^I^	10	10	10	10
THEORICAL COMPOSITION (% DM) **				
CP (%)	35.0	35.0	35.0	35.0
CL (%)	10.0	10.0	10.0	10.0
CHO (%)	48.9	47.0	46.7	46.8
ASH (%)	6.2	8.1	8.4	8.3
CA (%)	0.52	0.52	0.78	0.52
P (%)	0.66	1.16	1.16	1.16
CA/P	0.79	0.45	0.67	0.45

^a^ Fishmeal: CP 71.0%; CL 10.4%; Ash 19.1%; Ca 3.7%; P 2.7%; dm 92.2%. ^b^ Wheat meal: 18.8%; GB 1.2%; CHO 77.9%; Ash 2.1%; Ca 0.07%; P 0.36%; dm 91.5%. ^c^ Soybean meal: CP 45.1%; GB 9.0; CHO 38.8%; Ash 7.10%; Ca 0.36%; P 0.70%; dm 92.60%. ^d^ Wheat gluten: CP 84%; CHO 12.35%; Ash 3.65%; Ca 0.05%; P 0.17%; dm 93.00%. ^e^ MAP: Ash 100%; Ca 0.040%; P 26.476%; dm 99.45%. ^f^ SCP-2%, AQphos+: Ash 100%; Ca 12.158%; P 23.404; dm 98.70%. ^g^ MSP: Ash 100%; Ca 0.000%; P 24.024%; dm 99.90%. ^h^ Vitamin premix: Ash 100%; Ca 0.58%; P 0.645%. ^i^ Soybean lecithin: GB 100%; P 1.86%. ** Estimated according to the raw material of analysed composition.

**Table 2:** Composition of experimental diets expressed as % dry matter (Dm) *.

DIET	Ca (%)	P (%)	Ca/P	Na (%)	N (%)	CP ** (%)	Dm (%)
CONTROL	0.55	0.66	0.81	0.22	5.56	34.73	95.0
MAP	0.65	1.16	0.58	0.25	6.06	37.96	93.0
SCP-2%	0.80	1.16	0.72	0.27	5.68	35.52	92.9
MSP	0.56	1.16	0.49	0.55	5.60	35.03	94.2

* The data shown in the table correspond to triplicate analysis of diet samples once they have been extruded. ** Estimated as 6.25 × %N content.

**Table 3:** Apparent digestibility of diets (%) and inorganic phosphates used. Values are the mean *(n =* 4) ± standard error (SE). Different superscripts in the same row indicate significant statistical differences with *p* < 0.05. Newman-Keuls test.

Diets								
ADC (%)	Control	SE	MAP	SE	SCP-2%	SE	MSP	SE
Dry matter	75.0	2.7	77.2	1.4	74.7	1.4	75.3	2.0
Protein	67.2	1.0	67.1	2.3	66.6	1.1	66.3	1.9
P	75.4 a	1.2	84.3 b	0.3	84.3 b	0.4	86.0 b	0.4
Ca	34 a	3.3	40.3 ab	0.9	55.2 c	0.6	42.2 b	4.1
P source digestibility								
P			96.1 a	0.6	96.1 a	1.0	100.0 b	1.0
Ca					93.1	0.8		

The data in the table shows the mean of four tanks following a Latin square experimental design (*n* = 4) ± standard error of the mean (ES). Different superscripts in the same row indicate statistically significant differences with a *p* < 0.05. Newman-Keuls test.

**Table 4:** Growth, survival, and nutritional parameters of shrimp fed with diets with different inorganic phosphates.

DIETS	CONTROL	SE	MAP	SE	SCP-2%	SE	MSP	SE
INITIAL WEIGHT (G)	0.55	0.03	0.52	0.03	0.50	0.04	0.55	0.05
FINAL WEIGHT (G)	13.4	0.7	13.0	1.0	12.9	0.92	13.6	0.5
SGR (%/DAY) 1	3.33	0.05	3.35	0.06	3.38	0.07	3.35	0.06
SURVIVAL (%)	79.02	6.00	89.10	4.05	82.11	2.05	84.00	2.00
FIR 2	4.95	0.22	4.70	0.17	4.60	0.3	4.59	0.08
FCR 3	2.63	0.14	2.48	0.11	2.44	0.12	2.43	0.08

The data in the table show the mean of four tanks (*n* = 4) ± standard error of the mean (ES). Different letters in the same row indicate significant differences at *p* < 0.05. Newman-Keuls test. ^1^ Specific Growth Rate (% day^−1^), SGR: [ln (Average Final shrimp weight (g)) − ln (Average Initial shrimp weight (g))]/days of growth trial × 100. ^2^ Daily feed rate (g diet/day and 100 g shrimp biomass), FIR: Feed Intake (g)/Average Biomass (g) × 100/days. ^3^ Feed Conversion Rate, FCR: Feed Intake (g)/∆Biomass (g).

**Table 5:** Whole-body composition (% dm) at the beginning and at the end of the experimental trial and retention efficiencies per each diet.

	Initial	Control	SE	MAP	SE	SCP-2%	SE	MSP	SE
Dry Matter (%)	20.2	23.0	0.9	24.6	1.12	21.9	0.9	23.0	0.7
Crude Protein (%)	15.2	17.0	0.7	18.5	0.9	16.1	0.7	17.4	0.7
Ash (%)	3.11	3.36	0.18	3.38	0.13	3.32	0.11	3.45	0.15
P (%)	0.23	0.28	0.01	0.32	0.01	0.28	0.01	0.29	0.01
Ca (%)	0.64	0.74	0.02	0.73	0.03	0.71	0.02	0.70	0.02
NPV (%) 1		20.4	1.3	21.2	1.7	19.8	1.4	23.4	1.4
CaPV (%) 2		55.2 b	2.48	49.1 a	2.00	40.1 a	2.05	54.2 b	2.02
PPV (%) 3		17.1 b	0.9	12.7 a	1.12	11.4 a	0.9	11.3 a	0.9

The data in the table show the mean of four tanks (*n* = 4) ± standard error of the mean (ES). Different letters in the same row indicate significant differences at *p* < 0.05. Newman-Keuls test. ^1^ NPV = Nitrogen Productive Value (%), or N retention efficiency = Retained N (N of final shrimp (%) × Final biomass (g)) × 100 − (N of initial shrimp (%) × initial biomass (g))/N ingested (kg Feed ingested × % N). ^2^ CaPV = Productive Value of Ca (%), or Ca retention efficiency = Retained Ca (final fish Ca (%) × Final biomass (g)) × 100 − initial shrimp Ca × initial biomass (g)/Ca ingested. ^3^ PPV = Productive Value of Phosphorus (%), or P retention efficiency = Retained Phosphorus (P final shrimp (%) × Final Biomass (g)) × 100 − P initial shrimp (%) × Initial biomass (g)/Phosphorus ingested.

**Table 6:** Daily excretion of total ammoniacal nitrogen (N-TAN) and phosphorus (P-PO_4_^3−^) expressed in mg of N or P/100 g of shrimp per day *. The mean weight of the shrimp in the excretion test was 20–21 g.

	N-NAT	SEM	P-PO_4_^3−^	SEM
CONTROL	31.62	5.80	5.72	2.29
MAP	39.15	2.63	9.74	2.82
SCP-2%	33.34	2.30	9.71	1.17
MSP	36.40	6.11	11.53	3.02

* The data in the table show the mean of four tanks (*n* = 4) ± standard error of the mean (ES). Different superscripts in the same column indicate statistically significant differences with a *p* < 0.05. Newman-Keuls test.

## Data Availability

Data are contained within the article.
